# Clinical Course and Changes in High-Resolution Computed Tomography Findings in Patients with Idiopathic Pulmonary Fibrosis without Honeycombing

**DOI:** 10.1371/journal.pone.0166168

**Published:** 2016-11-09

**Authors:** Hiroyoshi Yamauchi, Masashi Bando, Tomohisa Baba, Kensuke Kataoka, Yoshihito Yamada, Hiroshi Yamamoto, Atsushi Miyamoto, Soichiro Ikushima, Takeshi Johkoh, Fumikazu Sakai, Yasuhiro Terasaki, Akira Hebisawa, Yoshinori Kawabata, Yukihiko Sugiyama, Takashi Ogura

**Affiliations:** 1 Department of Medicine, Jichi Medical University, Tochigi, Japan; 2 Department of Respiratory Medicine, Kanagawa Cardiovascular and Respiratory Center, Kanagawa, Japan; 3 Department of Respiratory Medicine and Allergy, Tosei General Hospital, Aichi, Japan; 4 Department of Respiratory Medicine, JR Tokyo General Hospital, East Japan Railway Company, Tokyo, Japan; 5 First Department of Internal Medicine, Shinshu University School of Medicine, Nagano, Japan; 6 Department of Respiratory Medicine, Toranomon Hospital, Tokyo, Japan; 7 Department of Respiratory Medicine, Japanese Red Cross Medical Center, Tokyo, Japan; 8 Department of Radiology, Kinki Central Hospital of Mutual Aid Association of Public School Teachers, Hyogo, Japan; 9 Department of Diagnostic Radiology, Saitama Medical University International Medical Center, Saitama, Japan; 10 Department of Analytic Human Pathology, Nippon Medical School, Tokyo, Japan; 11 Department of Clinical Research, National Hospital Organization Tokyo National Hospital, Tokyo, Japan; 12 Division of Diagnostic Pathology, Saitama Prefectural Cardiovascular and Respiratory Center, Saitama, Japan; Imperial College London, UNITED KINGDOM

## Abstract

Some patients with idiopathic pulmonary fibrosis (IPF) do not have honeycombing on high-resolution computed tomography (HRCT) at their initial evaluation. The clinical course and sequential changes in HRCT findings in these patients are not fully understood. We reviewed the cases of 43 patients with IPF without honeycombing on initial HRCT from institutions throughout Japan. All patients were diagnosed with IPF based on a surgical lung biopsy. Multidisciplinary discussions were held five times between 2011 and 2014, to exclude alternative etiologies. We evaluated the sequential changes in HRCT findings in 30 patients with IPF. We classified these 30 patients into three groups based on their HRCT patterns and clarified the clinical characteristics and prognosis among the groups. The patterns of all 30 patients on initial HRCT corresponded to a possible usual interstitial pneumonia (UIP) pattern which was described in the 2011 International Statement. On long-term follow-up (71.0±38.7 standard deviation [SD] months), honeycombing was seen in 16 patients (53%, the HoneyCo group); traction bronchiectasis or cysts without honeycombing was observed in 12 patients (40%, the NoHoneyCo group), and two patients showed no interval change (7%, the NoChange group) on HRCT. The mean survival periods of the HoneyCo and NoHoneyCo groups were 67.1 and 61.2 months, respectively (p = 0.76). There are some patients with IPF whose conditions chronically progress without honeycombing on HRCT. The appearance of honeycombing on HRCT during the follow-up might not be related to prognosis.

## Introduction

In 2000, the American Thoracic Society (ATS) and European Respiratory Society (ERS) in collaboration with the American College of Chest Physicians published an international consensus statement on the diagnosis and management of idiopathic pulmonary fibrosis (IPF) [1], and since then accumulated data and observations of the radiological patterns of IPF have contributed to new guidelines. IPF is characterized by progressive worsening of pulmonary function and is associated with a poor prognosis [2]. Several retrospective longitudinal studies suggest that the median survival of IPF patients is 3–5 years after diagnosis [3–6], which is a worse rate than those of several types of cancer [7]. The majority of patients with IPF show slow but steady worsening (“slow progression”) [8], while the natural course of IPF appears to be heterogeneous [2], [9–10].

According to the 2011 ATS/ERS/Japanese Respiratory Society (JRS)/Latin American Thoracic Association (ALAT) guideline, the diagnosis of IPF should be based on the findings obtained using the combination of high-resolution computed tomography (HRCT) and surgical lung biopsy with a formal multidisciplinary discussion among the treating pulmonologist, radiologist, and pathologist [8]. If the patient’s HRCT findings meet the criteria for the usual interstitial pneumonia (UIP) pattern— in which honeycombing is critical for making the diagnosis of the UIP pattern — a surgical lung biopsy is unnecessary. This IPF guideline thus placed great importance on honeycombing on HRCT. If honeycombing is absent, but the imaging features otherwise meet the criteria for the UIP pattern, the imaging features are regarded as representing a ‘possible UIP pattern,’ and the surgical lung biopsy pattern must be a UIP pattern or a probable UIP pattern to make a definitive diagnosis of IPF [8]. However, the clinical course and the sequential changes in HRCT findings of these IPF patients are not fully understood. It is yet to be ascertained whether a possible UIP pattern progresses to a UIP pattern on HRCT.

The purposes of the present study were to (1) retrospectively assess the sequential changes in the HRCT pattern in IPF patients who did not show honeycombing on their initial HRCT, and (2) clarify these patients’ clinical outcomes.

## Patients and Methods

**Fig 1:**
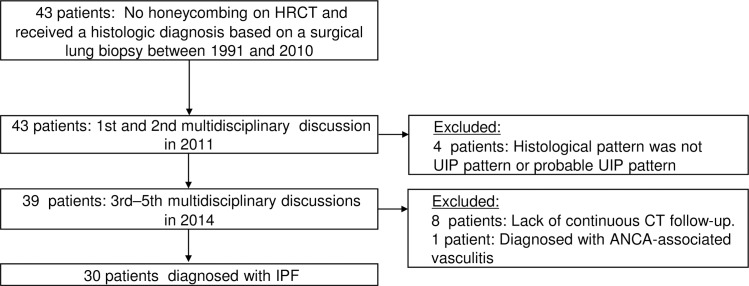
Establishing the diagnosis of idiopathic pulmonary fibrosis (IPF) by multidisciplinary discussions.

Institutional review board approval was obtained for this retrospective study (Bioethics Committee for Clinical Research A, Jichi Medical University Hospital; A15-180). We reviewed the cases of 43 patients with IPF without honeycombing on their initial HRCT from 14 institutions throughout Japan. All patients underwent a surgical lung biopsy between 1991 and 2010 (S1 Table). The initial multidisciplinary discussions of the 43 cases were held at the 11th Tokyo Diffuse Lung Research Meeting in 2011. Thirty-nine patients were histologically diagnosed as showing a UIP pattern or a probable UIP pattern, and the other four patients were excluded from the present study because they showed another histological pattern. Three years later, at the 15th Tokyo Diffuse Lung Research Meeting in 2014, we evaluated the changes in the 39 patients’ HRCT findings, their serological findings, and the changes in pulmonary functions such as forced vital capacity (FVC) and diffusing capacity for carbon monoxide (DLco). The cases of eight patients were excluded due to a lack of sequential HRCT scans. One patient was excluded because the patient was considered to have anti-neutrophil cytoplasmic antibody-associated vasculitis instead of IPF. The series of procedures for patient accrual to the study is shown in Fig 1. A final total of 30 patients were given the diagnosis of IPF in 2014, and their cases were enrolled in this study.

The first study outcome was our classification of these 30 patients into three groups based on their HRCT patterns. Our second goal was to clarify the clinical characteristics and prognosis among these groups.

### CT scan evaluation

**Fig 2:**
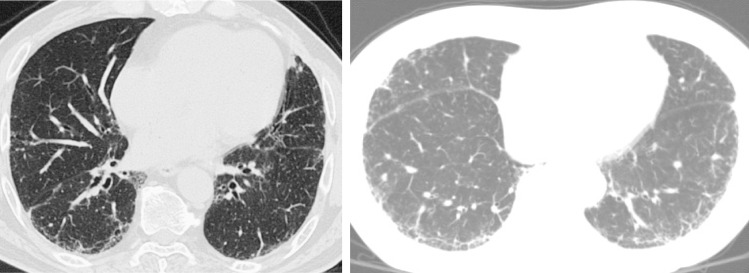
HRCT images of two patients with representative HRCT scans, showing reticular abnormalities and/or subpleural irregularities without honeycombing.

We reviewed the 30 patients’ initial HRCT scans obtained several months before the surgical biopsies. The initial HRCT scan findings in the patients without honeycombing were discussed in 2011. All available HRCT images were independently evaluated by two chest radiologists with 35 and 27 years of experience, respectively. The predominance of several pulmonary abnormalities such as ground glass opacities, reticular shadows, honeycombing, traction bronchiectasis and cysts was evaluated. The diagnoses based on HRCT findings of a possible UIP pattern were based on the 2000 guidelines [1]. Final decisions regarding the findings and diagnosis were reached by consensus. Any discrepancies were resolved by repeated multidisciplinary discussions. The HRCT images of two representative patients with a possible UIP pattern on HRCT are shown in Fig 2. Continuous follow-up HRCT images were obtained after 2011. All available HRCT findings were also evaluated by the same pulmonary radiologists in 2014.

### Histological evaluation

All patients had no honeycombing on the initial HRCT scans, and each patient underwent a surgical lung biopsy between 1991 and 2010. The histological findings were discussed in 2011. Tissue samples were scored by two thoracic pathologists with 37 and 33 years of experience, respectively. Diagnoses from pathological findings were based on the 2011 ATS/ERS/JRS/ALAT guidelines [8]. The histopathological features of a UIP pattern included evidence of marked fibrosis/architectural distortion with or without honeycombing in a predominantly subpleural/paraseptal distribution of patchy involvement of lung parenchyma by fibrosis, the presence of fibroblast foci, and the absence of features against a diagnosis of UIP or suggesting an alternate diagnosis. The histopathological features of a probable UIP pattern allow the absence of either patchy involvement or fibroblastic foci, but not both.

### Statistical analysis

A p-value <0.05 was considered significant in all tests. Statistical analyses were performed using SAS software ver. 5.0 (SAS Institute, Cary, NC, USA). The survival rates of the patients in the three groups were compared using the log rank test and are shown as Kaplan-Meier curves. We analyzed the interobserver variation of the existence of various abnormalities and HRCT pattern using the kappa (κ) statistic. The interobserver agreement was classified as follows: poor, κ  =  0–0.20; fair, κ  =  0.21–0.40; moderate, κ  =  0.41–0.60; good, κ  =  0.61–0.80; and excellent, κ  =  0.81–1.00.

## Results

The agreements of the two observers regarding the HRCT interpretation were good to excellent (Kappa value >0.7). The patients’ characteristics are summarized in Table 1. All 30 patients (22 men and eight women) were diagnosed with a possible UIP pattern on the initial HRCT scan. They were also diagnosed with a UIP pattern or a probable UIP pattern based on the initial histopathological evaluation. The mean age of the patients was 64.5 ± 6.3 standard deviation (SD) years (range 50–79 years). The mean percentage of predicted values for FVC (%FVC) and DLco (%DLco) were 88.3% and 80.4%, respectively.

**Table 1:** Baseline characteristics of patients with a UIP pattern or a probable UIP pattern based on histopathological criteria and a possible UIP pattern based on HRCT criteria without honeycombing.

	Total (n = 30)	HoneyCo (n = 16)	NoHoneyCo (n = 12)	NoChange (n = 2)
Age, yr	64.5 ± 6.3			
50–59	7	3	4	
60–69	17	10	5	2
≥70	6	3	3	
Male: female	22: 8	14: 2	7: 5	1: 1
MRC grade:				
0	10	5	4	1
1	5	3	2	
2	13	6	6	1
3	2	2		
Smoking history:				
Never smoker	13	5	7	1
Ex-smoker	11	8	3	
Current smoker	6	3	2	1
Lung function:				
FVC % pred	88.3 ± 17.2	84.6 ± 16.8	91.0 ± 17.0	101.7 ± 21.5
DLco % pred	80.4 ± 22.4	77.1 ± 23.7	85.1 ± 22.4	77.2± 13.9
PaO_2_ (%)	84.5 ± 10.4	88.3 ± 10.7	81.9 ± 7.7	69.9 ± 4.6
KL-6 (U/mL)	1082.1 ± 554.1	1221.4 ± 564.0	978.6 ± 534.5	589.0 ± 256.0
SP-D (ng/mL)	284.0 ± 130.1	268.6 ± 125.3	322.7 ± 133.8	

MRC: Medical Research Council

FVC % pred: A percentage of those predicted of Forced Vital Capacity

DLco % pred: A percentage of those predicted of diffusing capacity of the lungs for carbon monoxide

PaO_2_: Partial pressure of oxygen in arterial blood

KL-6: Krebs von den Lungen-6

SP-D: Surfactant protein D.

**Fig 3:**
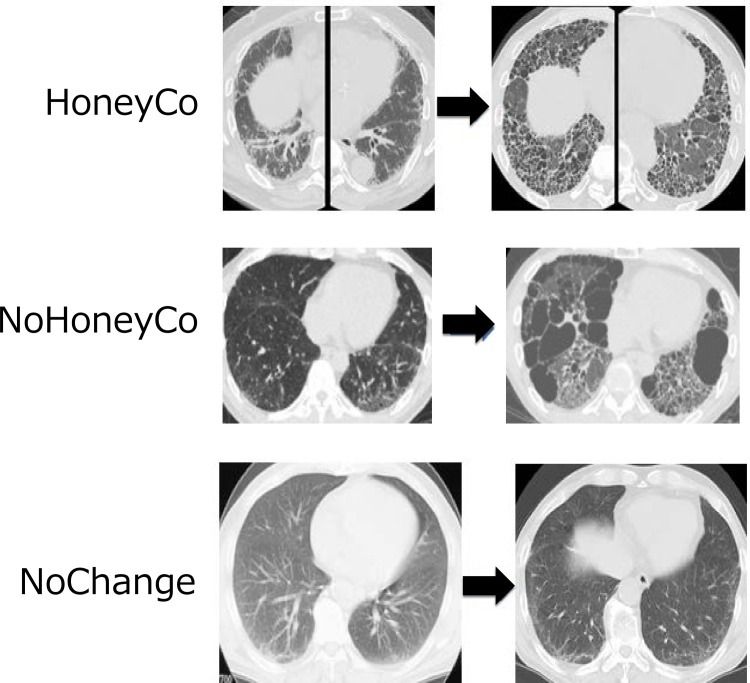
Representative HRCT scans from the three patient groups. HoneyCo: Honeycombing became evident on HRCT scan. NoHoneyCo: Traction bronchiectasis or cysts became evident on HRCT scan. NoChange: No significant change in HRCT findings.

All 30 patients with IPF were classified based on the sequential changes in HRCT. The mean follow-up duration from the initial HRCT was 71.0 ± 38.7 (SD) months (range 16–138 months). We classified the patients into one of the following three groups: HoneyCo group; NoHoneyCo group or NoChange group. The HoneyCo group was the patients in whom honeycombing emerged on subsequent HRCT scans after the initial evaluation. The NoHoneyCo group was patients in whom honeycombing did not emerge, but traction bronchiectasis or a cyst emerged on subsequent HRCT scans after the initial evaluation. The NoChange group included patients who did not have any significant changes on subsequent serial HRCT scans after the initial evaluation (Fig 3). Sixteen patients (53%) were classified into the HoneyCo group, 12 (40%) into the NoHoneyCo group, and the remaining two patients (7%) were classified into the NoChange group.

**Fig 4:**
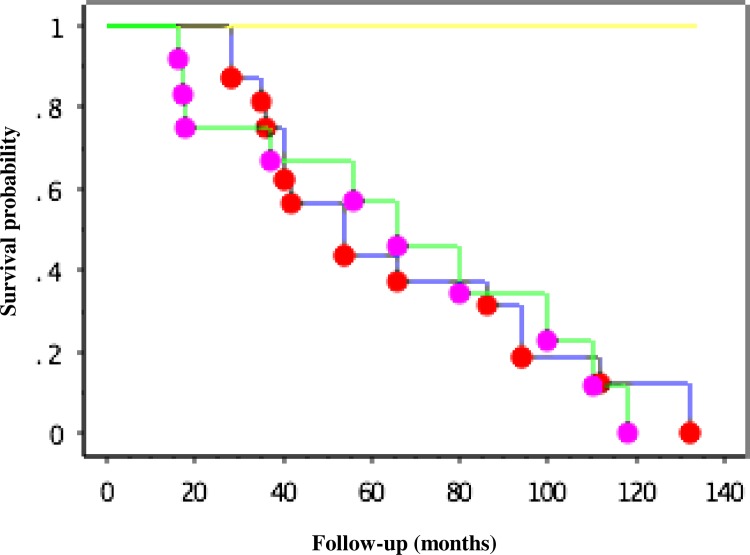
Kaplan-Meier survival curves where honeycombing appeared on subsequent HRCT scans after initial evaluation (n = 16, blue line, the HoneyCo group), patients in whom honeycombing was not seen but traction bronchiectasis or cysts appeared on subsequent computed tomography scans after initial evaluation (n = 12, green line, the NoHoneyCo group), and patients with no change in HRCT findings after long-term follow-up (n = 2, yellow line, the NoChange group).

The baseline characteristics among these three groups were not significantly different, although the HoneyCo group had a tendency to have slightly low %FVC and %DLco values. The values of FVC decline in the HoneyCo and NoHoneyCo groups were −274 ± 181 (SD) ml/year (range −79 to −680 ml/year) and −395 ± 478 (SD) ml/year (range −101 to −1440 ml/year) respectively, which are not significantly different (p = 0.92). Patients in the NoHoneyCo group did not form honeycombing, but the long-term observation of serial HRCT images revealed that their traction bronchiectasis or cysts progressed to an appearance suggestive of a nonspecific interstitial pneumonia (NSIP) pattern or an appearance that was inconsistent with a UIP pattern. The Kaplan-Meier survival curves of the three groups are shown in Fig 4. The mean survivals of the HoneyCo group patients was 67.1 months, and that of the NoHoneyCo patients was 61.2 months; the survival periods were not significantly different (p  =  0.76).

## Discussion

The results of the present study showed that IPF patients who do not have honeycombing on the initial HRCT scans can be classified patterns of the sequential changes in HRCT into three groups. One group of IPF patients showed the typical HRCT pattern of IPF; i.e., the appearance of honeycombing. Another group showed an atypical HRCT pattern of IPF; i.e., the appearance of traction bronchiectasis or cysts without honeycombing. Our analysis of the prognoses of these two groups indicated that the presence or absence of honeycombing on HRCT during the follow-up is not related to prognosis. Our third, small patient group showed stable findings on HRCT, which can be expected to suggest a good prognosis.

It was recently reported that some patients with IPF have little or no radiological evidence of honeycombing at the time of diagnosis [11], but there have been no long-term longitudinal studies of such patients. The clinical course and the sequential changes in HRCT findings of these IPF patients are not fully understood, and it is yet to be ascertained whether a possible UIP pattern progresses to a UIP pattern on HRCT. The present study was a first attempt to observe temporal HRCT changes in these patients.

According to the 2011 international (ATS/ERS/JRS/ALAT) statement, the key finding on HRCT to differentiate between possible UIP/IPF and a UIP/IPF pattern is the absence or presence of honeycombing. Honeycombing is seen on HRCT as clusters of cystic spaces, typically with diameters on the order of 3–10 mm with 1–3 mm wall thickness, but they may be up to 25 mm in diameter [12]. Previous studies examined the correlation between CT scan and histologic findings in IPF patients. Schettino *et al*. reported that the honeycomb pattern shown on HRCT scans was correlated with that determined by histology (r  =  0.71; p<0.01, kappa 0.3) [13]. Kazerooni *et al*. reported that CT fibrosis scores, determined by the ratio of honeycombing to the lobe, strongly correlate with pathology fibrosis scores [14].

The first major finding of the present study is that some possible UIP/IPF patients did not develop a definite UIP/IPF pattern but developed traction bronchiectasis or cysts that finally progressed to an appearance that was suggestive of NSIP or inconsistent with a UIP pattern on HRCT. Although we were unable to find any studies in the literature in which possible UIP/IPF patients developed an appearance suggestive of NSIP, there are some reports that NSIP patients developed a UIP pattern on CT. Akira *et al*. reported that the HRCT appearance of NSIP progressed to an appearance suggestive of IPF because ground-glass opacity and consolidation decreased whereas the coarseness of fibrosis increased on the follow-up HRCT scans in most NSIP patients [15]. Silva *et al*. reported that 28 percent of patients with initial HRCT findings suggestive of NSIP progressed to findings suggestive of IPF [16].

Another research group reported their comparison between CT patterns and pathologic diagnoses: Sumikawa *et al*. noted that 21 of 56 (38%) patients with an NSIP pattern at CT were classified as having pathologic UIP; however, the sequential changes in HRCT findings of these patients were not described in that study [17]. Though several past studies suggested that wider honeycombing is one of the HRCT findings suggesting the diagnosis of IPF [18], [19] and recent IPF guidelines might set a high value on HRCT patterns to diagnose IPF, honeycombing on HRCT may not be essential for diagnosing IPF.

A second important finding of the present study is that was no significant difference in the prognoses of the IPF patients who did or did not eventually form honeycombing as shown by HRCT. The mean survival times of these patients were 67.1 and 61.2 months, respectively. IPF has an unknown etiology and a very poor prognosis. Raghu *et al*. demonstrated that IPF patients aged ≥65 years were living longer in 2011 than they were 10 years before [20], while a 2015 study showed that the median survival of IPF patients is 3–5 years from the time of diagnosis [6]. A baseline factor of “extent of honeycombing on HRCT” was associated with an increased risk of mortality, although it is unknown whether the presence of these features constitutes a subpopulation of patients with end-stage IPF, because of the variability in the natural history of the disease [8].

Bando *et al*. reported that the median survival time of IPF patients in Japan from the initial visit was 69 months [21], which is similar to our present findings. This might indicate that the presence of honeycombing on the initial HRCT scan does not directly correlate with the prognosis of IPF patients. A recent randomized, double-blind, placebo-controlled IPF study also supported the results of the present study, showing that 298 IPF patients with honeycombing on their initial HRCT had a −225.7 ml/yr decline in FVC values, whereas 125 patients without honeycombing on HRCT had a −221.0 ml/yr decline in FVC; this difference was not significant [22]. These two groups were placebo groups in the study, which might be equivalent to the natural history of IPF [22].

Third, we found that a few patients with possible UIP/IPF did not progress over the long-term follow-up. According to the 2011 international statement, the natural clinical course of patients with IPF is variable, and the course can be described as “slow progression,”“stable,” and”rapid progression,” [8] but the percentage of patients in each category is unclear. In the present study, two of the 30 (7%) patients with possible UIP/IPF showed stable findings on HRCT and were classified as the NoChange group.

This study had some limitations. It was retrospective and included only patients who underwent a surgical biopsy. The decision whether or not to perform a surgical biopsy was made at each institution, and this may have led to selection bias. In addition, we calculated the mean survival time from the diagnosis of IPF, which also may have differed by each institution. Other factors that may affect the lungs such as smoking, dust inhalation and medications were not taken into account in this study. It may also be difficult to draw any firm conclusions because there were only 30 patients.

In conclusion, in our patients with pathologically proven UIP/IPF, the presence or absence of honeycombing on HRCT was not related to prognosis. Although recent IPF guidelines state that the HRCT pattern is a factor in making the diagnosis of IPF, honeycombing on HRCT might not be essential to establish this diagnosis.

## Supporting Information

S1 Table List of 43 patients in 2011. (TIF)

## Abbreviations

ALAT: Latin American Thoracic Association
ATS: American Thoracic Society
DLco: diffusing capacity for carbon monoxide
ERS: European Respiratory Society
FVC: forced vital capacity
HRCT: high-resolution computed tomography
IPF: idiopathic pulmonary fibrosis
JRS: Japanese Respiratory Society
NSIP: nonspecific interstitial pneumonia
UIP: usual interstitial pneumonia
