# Supplementary figures and images for: Clinical Course and Changes in High-Resolution Computed Tomography Findings in Patients with Idiopathic Pulmonary Fibrosis without Honeycombing
Source: PLoS One. 2016 Nov 9;11(11):e0166168. doi: 10.1371/journal.pone.0166168 (PMC5102464; doi:10.1371/journal.pone.0166168)

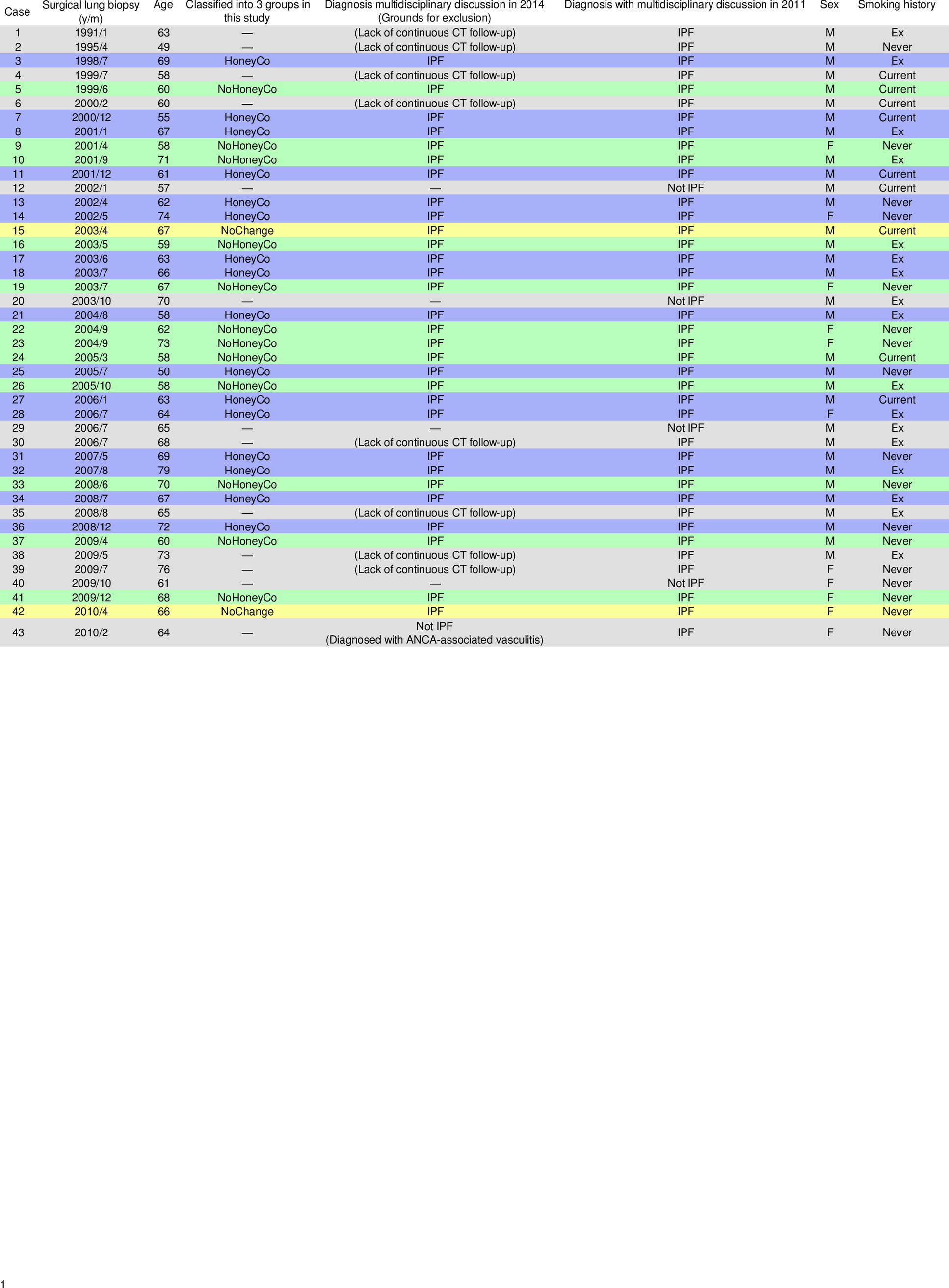

Supplement: S1 Table — (TIF) [file pone.0166168.s001.tif]
